# Adaptation of Brain Functional and Structural Networks in Aging

**DOI:** 10.1371/journal.pone.0123462

**Published:** 2015-04-15

**Authors:** Annie Lee, Nagulan Ratnarajah, Ta Anh Tuan, Shen-Hsing Annabel Chen, Anqi Qiu

**Affiliations:** 1 Department of Biomedical Engineering, National University of Singapore, Singapore 117576, Singapore; 2 Division of Psychology, Nanyang Technological University, Singapore 637332, Singapore; 3 Clinical Imaging Research Center, National University of Singapore, Singapore 117456, Singapore; 4 Singapore Institute for Clinical Sciences, the Agency for Science, Technology and Research, Singapore 117609, Singapore; & National Laboratory of Pattern Recognition, CHINA

## Abstract

The human brain, especially the prefrontal cortex (PFC), is functionally and anatomically reorganized in order to adapt to neuronal challenges in aging. This study employed structural MRI, resting-state fMRI (rs-fMRI), and high angular resolution diffusion imaging (HARDI), and examined the functional and structural reorganization of the PFC in aging using a Chinese sample of 173 subjects aged from 21 years and above. We found age-related increases in the structural connectivity between the PFC and posterior brain regions. Such findings were partially mediated by age-related increases in the structural connectivity of the occipital lobe within the posterior brain. Based on our findings, it is thought that the PFC reorganization in aging could be partly due to the adaptation to age-related changes in the structural reorganization of the posterior brain. This thus supports the idea derived from task-based fMRI that the PFC reorganization in aging may be adapted to the need of compensation for resolving less distinctive stimulus information from the posterior brain regions. In addition, we found that the structural connectivity of the PFC with the temporal lobe was fully mediated by the temporal cortical thickness, suggesting that the brain morphology plays an important role in the functional and structural reorganization with aging.

## Introduction

Converging evidence from task-based functional magnetic resonance imaging (fMRI) studies suggests pronounced aging effects on functional activities in the prefrontal cortex (PFC). Older adults exhibit more PFC activity ipsilaterally or bilaterally as compared to their younger counterparts in various tasks [1–3]. The age-related increase in bilateral frontal activation seemed to suggest that older adults were working harder and engaging in more distributed brain regions. Moreover, frontal processing in older adults appeared to be less specialized through a tendency to engage additional frontal regions, while frontal processing in young adults only involved specific PFC regions across multiple cognitive tasks, such as working memory [3, 4], episodic memory [1, 2], attentional and perceptual tasks [5, 6], and semantic tasks [7].

In contrast, posterior regions of the brain often show age-related reduction in functional responses and dedifferentiation to stimuli [2, 8–14]. Particularly, the ventral visual cortex became less functionally distinct in the sense that it became less selective to visual inputs in older adults [15, 16]. In young adults, the fusiform and lateral occipital regions are specialized for facial and object recognition, while the parahippocampal and lingual regions are specialized for encoding new perceptual information about the appearance and layout of scenes [15]. However, in older adults, these brain regions tend to lose these functional specificities. This decrease in neural specificity was also thought of as dedifferentiation such that a given region that responds selectively in young adults will respond to a wider array of inputs in older adults.

Interestingly, age-related dedifferentiation of functional processes in the ventral visual pathway could be compensated by an age-related increase in PFC functional activation [15–21]. Additional recruitment of the PFC corresponds to an attempt to compensate for reduced functional specificities of posterior regions in older adults [22]. Functional connectivity studies based on memory tasks suggested that stronger functional connectivity among the posterior brain regions is shown in young adults but stronger connectivity between the posterior regions and PFC is shown in older adults [10, 14, 17]. Davis et al. [9] further confirmed this shift from posterior brain activations to anterior activations, and suggested that the increased frontal activation with age is in response to deficient ventral visual and sensory activations. Overall, there is growing evidence that the additional work of the frontal sites may be a broad response to decreased efficiency of neural processes in perceptual areas of the brain [23]. In other words, dedifferentiation in the posterior brain may play as an impetus for the PFC compensation in normal aging.

Though the aforementioned findings have been constructive in aging studies, controversial results were also found. For instance, Lidaka et al. revealed that young adults showed bilateral PFC activity while older adults showed unilateral PFC activity during associative learning of the concrete-unrelated or abstract pictures [24]. Duveme et al. showed that additional frontal activity was revealed only in low-performing older adults [25]. These inconsistent results may be partly due to confounding factors, such as task difficulty and subject’s incompliancy associated with task-based fMRI [26, 27].

In recent years, resting-state fMRI (rs-fMRI) has become influential, as it requires a minimal cognitive burden on participants and relatively little time in the scanner compared to task-based fMRI. Unlike task-based fMRI, rs-fMRI cannot be used to reveal functional activations in response to sequential external stimuli during cognitive tasks. However, rs-fMRI enables a summarization of complex patterns of brain functional organization [28–30]. It has been well used to explore age-related changes in default-mode network (DMN) [30–38]. However, there are limited investigations into whether aged-related changes in the PFC and posterior regions of the brain observed using task-based fMRI can be replicated at the level of functional connections examined using rs-fMRI.

Likewise, little is known if aforementioned changes can be observed using structural MRI and diffusion tensor imaging (DTI) techniques. Though examination of structural brain network in conjunction with functional brain network could provide complementary findings on how the brain adapted to age-related changes, a large body of aging research on structural networks focused on differentiation of pathological aging from normal aging as well as age-related changes in white matter integrity [39]. Only recently, Gong et al. employed DTI and structural network analysis and revealed that the frontal and temporal lobes showed an age-related increase in regional efficiency in terms of information transfer, while the parietal and occipital lobes showed an age-related decrease in regional efficiency [40]. However, this study did not examine age effects on structural connectivity between the PFC and posterior regions of the brain in order to link structural network findings with the aforementioned age-related changes in functional activations of PFC and posterior regions in the aging brain.

In the present study, we hypothesize that functional networks examined using rs-fMRI and structural networks accessed using high angular resolution diffusion imaging (HARDI) can demonstrate age-related compensatory changes in the PFC and posterior regions of the brain at the level of their connections. In particular, we hypothesize that the functional and structural connectivity of the PFC with the posterior regions of the brain increases as age increases. Such age effects could be mediated by the functional and structural connectivity among the posterior regions of the brain. Given well-known knowledge on age-related brain atrophy, we also hypothesize that the above age effects may also partially be mediated by brain atrophy. Hence, we employed rs-fMRI, high angular resolution diffusion imaging (HARDI), and graph analysis techniques to examine i) age effects on structural and functional connectivity of the PFC with posterior regions of the brain; ii) mediation effects of structural and functional connectivity among the posterior regions of the brain on age-related changes in structural and functional connectivity of the PFC; iii) mediation effects of brain atrophy on age-related changes in structural and functional connectivity of the PFC. Unlike previous studies where analyses were restricted to comparing two age groups (young versus old) [9, 30, 32, 33, 36] or with a small number of subjects across a wide age range [37, 41], we examine age-related connectivity based on 173 subjects aged from 21 to 80 years old (evenly distributed across this age range) to establish a more comprehensive understanding of brain network changes. Moreover, we apply HARDI to examine structural networks to overcome the well-known limitation of DTI, where only one dominant fiber orientation at each location is revealed. Between one and two thirds of the voxels in the human brain white matter are thought to contain multiple fiber bundles crossing each other [42]. It has been shown that accurate fiber estimates can be obtained from HARDI data, further validating its usage in brain studies [43]. In addition, we use cortical thickness as an indicator of brain morphological measures in our functional and structural network analysis. This is to control for the possible confound of age-related reduction in cortical thickness [44, 45], which has not been accounted for in most of imaging aging studies so far.

## Methods

### Subjects

This study was approved by the National University of Singapore Institutional Review Board and all participants provided written informed consent prior to participation.

Two hundred and fourteen healthy Singaporean Chinese volunteers aged 21 to 80 years old were recruited (males: 93; females: 121) for this study. Volunteers with the following conditions were excluded: (1) major illnesses/surgery (heart, brain, kidney, lung surgery); (2) neurological or psychiatric disorders; (3) learning disability or attention deficit; (4) head injury with loss of consciousness; (5) non-removable metal objects on/in the body such as cardiac pacemaker; (8) diabetes or obesity; (9) a Mini-Mental State Examination (MMSE) score of less than 24 [46]. This study only included 173 subjects who were right handed and completed both functional and structural scans. Subjects’ characteristics are reported in Table 1.

**Table 1:** Subject characteristic.

Age	20s *mean (SD)*	30s *mean (SD)*	40s *mean (SD)*	50s *mean (SD)*	60s above *mean (SD)*
N	32	24	27	42	48
Age	25.6(2.22)	34(2.54)	44.8(2.68)	54.8(3.11)	67.4(4.83)
Female, %	56	50	63	60	71

SD-standard deviation.

### Data acquisition

MRI scans were acquired in a 3T Siemens Magnetom Trio Tim scanner using a 32-channel head coil at the Clinical Imaging Research Centre of the National University of Singapore. The image protocols were (i) high-resolution isotropic T_1_-weighted Magnetization Prepared Rapid Gradient Recalled Echo (MPRAGE; 192 slices, 1mm thickness, sagittal acquisition, field of view 256 x 256 mm, matrix = 256 x 256, repetition time = 2300ms, echo time = 1.90ms, inversion time = 900ms, flip angle = 9°); (ii) isotropic axial resting-state functional MRI imaging protocol (single-shot echo-planar imaging; 48 slices with 3 mm slice thickness, no inter-slice gaps, matrix = 64 × 64, field of view = 192 x 192 mm, repetition time = 2300ms, echo time = 25ms, flip angle = 90°, scanning time = 6 min); (iii) High angular resolution diffusion imaging protocol (HARDI, single-shot double-echo EPI sequence; 48 slices with 3 mm slice thickness, no inter-slice gaps, matrix = 84 × 84, field of view = 256 x 256 mm, repetition time = 6800ms, echo time = 85ms, flip angle = 90°, 11 images without diffusion weighting, 91 diffusion weighted images with b-value = 1150s/mm^2^, scanning time = 12 min). During the rs-fMRI scan, the subjects were asked to close their eyes.

### Data preprocessing and brain network construction

FreeSurfer was employed to parcellate the cortex in the subject’s native brain space based on surface-based registration and anatomical prior [47]. These labels were propagated to the volume, which was used in subsequent fMRI analysis. In contrast, labeling the cortex based on Automated Anatomical Labeling (AAL) atlas requires relatively accurate mapping of cortical regions between AAL atlas and the subject’s images, which has been shown less superior to surface-based mapping [48].

#### Structural data

**Fig 1:**
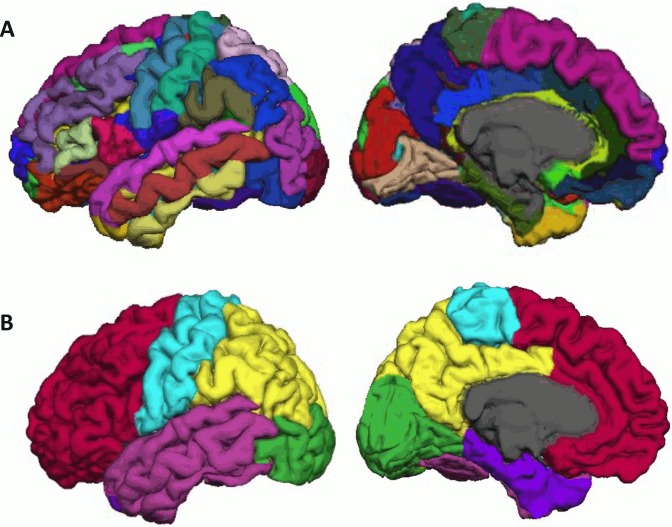
Brain parcellation. Panel (A) shows the cortical parcellation of the brain. Individual structures are coded in different color. Panel (B) shows the grouping of anatomical structures into the prefrontal cortex (red), motor and sensory cortex (cyan), parietal cortex (yellow), lateral temporal cortex (magenta), medial temporal lobe (purple), occipital cortex (green), and striatum-thalamic region (blue).

For the T_1_-weighted image, FreeSurfer was used to segment the cortical and subcortical regions, compute the cortical thickness and the cortical parcellation. Briefly, a Markov random field (MRF) model was used to label each voxel in the T_1_-weighted image as gray matter (GM), or white matter (WM), or CSF [49]. The T_1_-weighted images were fully automatically analyzed with manual quality check and propoer modification based on the instruction given on https://surfer.nmr.mgh.harvard.edu/fswiki. The most frequent modification included removal of the brain skull. Cortical inner surface was constructed at the boundary between GM and WM and then propagated to its outer surface at the boundary between GM and CSF. The cortical thickness was measured as the distance between the corresponding vertices on the inner and outer surfaces [50] and represented on the inner surface. The cortical surface of each hemisphere was parcellated in 36 anatomical regions (Table 2 and Fig 1A) in the rs-fMRI and HARDI analyses below.

**Table 2:** The grouping of anatomical structures.

Anatomical Structure Groups					
Prefrontal	Motor and Sensory Cortex	Occipital	Lateral Temporal	Medial Temporal	Parietal
superior frontal	precentral	lateral occipital	superior temporal	parahippocampus	superior parietal
caudal middle frontal	paracentral	lingual gyrus	transverse temporal	temporal pole	supramarginal
rostral middle frontal	postcentral gyrus	cuneus	middle temporal	entorhinal	inferior parietal
pars opercularis	insular		banks of superior temporal	hippocampus	pericalcarine
pars triangularis			fusiform	amygdala	precuneus
pars orbitalis			inferior temporal		posterior cingulate
lateral orbitofrontal					
medial orbitofrontal					
frontal pole					
anterior cingulate					

#### Rs-fMRI

**Fig 2:**
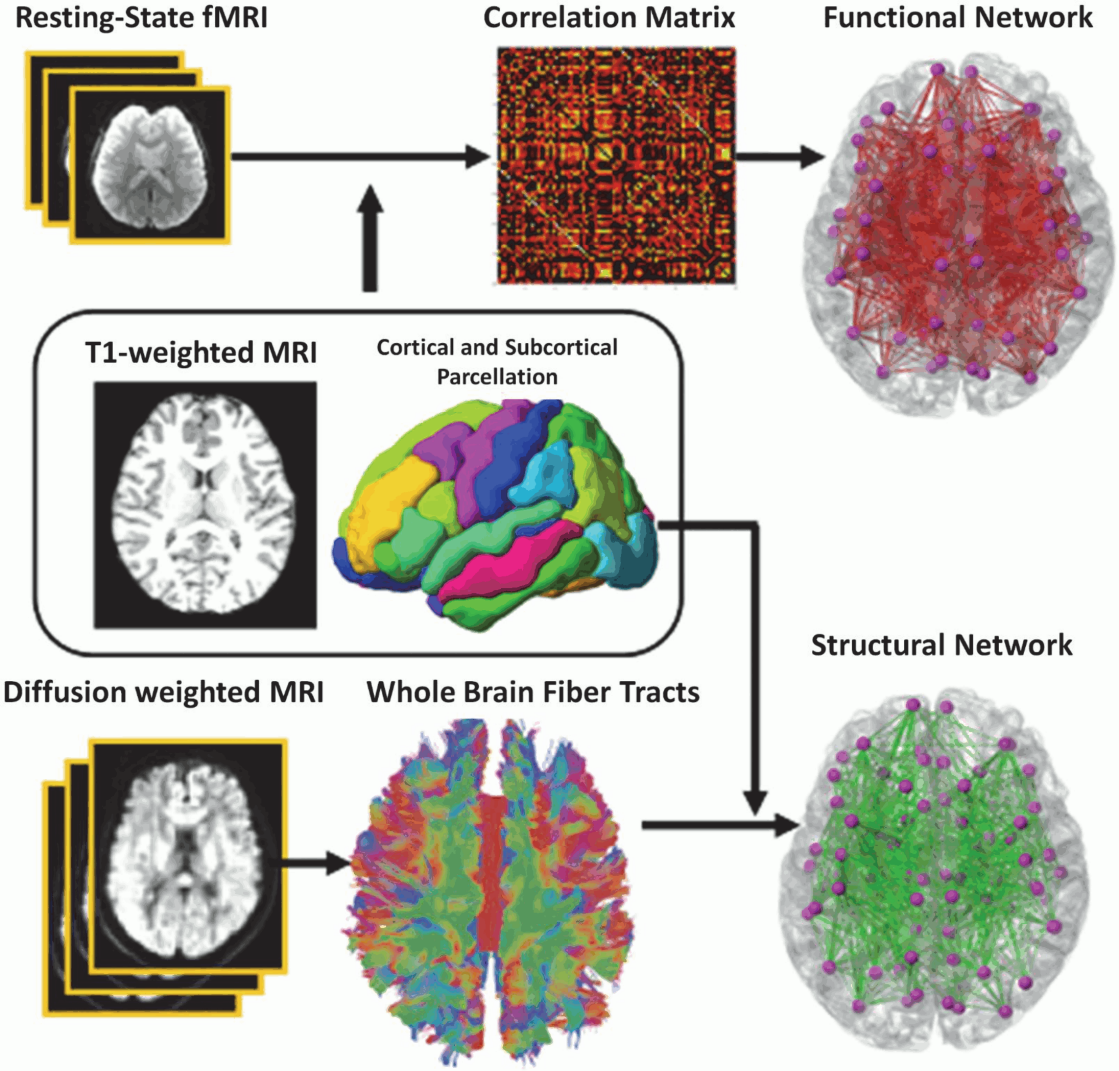
A schematic diagram of the functional and structural network analysis.

We employed SPM8 to preprocess the rs-fMRI data with slice timing, motion correction, skull stripping, band-pass filtering (0.01–0.08 *Hz*) and grand mean scaling of the data (to whole brain modal value of 100). To quantify the quality of rs-fMRI data in terms of head motion, displacement due to motion averaged over the image volume was calculated for individual subjects. Its mean and standard deviation were respectively 0.05 mm and 0.04 mm among all the subjects used in this study. The head motion was independent of age (p>0.05). Then, the rs-fMRI signals due to effects of nuisance variables, including six parameters obtained by motion correction, ventricular and white matter signals after band-pass filtering and grand-mean scaling were removed [51–54]. Subsequently, the fMRI data were transferred to the corresponding T_1_-weighted image. For functional network analysis, time series in each ROI defined using the T_1_-weighted data mentioned above (Fig 1A and Table 2) were first computed by averaging the signal of all voxels within individual ROIs. The functional connectivity of each subject was characterized using an 72 x 72 symmetric weighted matrix *W*_*ij*_ *W*_*i j*_ and the weight was computed using Pearson correlation analysis on the time series of regions *i* and *j*. The data processing of the rs-fMRI is summarized in Fig 2.

#### HARDI

For each subject, DWIs were first corrected for motion and eddy current distortions using affine transformation to the image. We followed the procedure detailed in Huang et al. (2008) to correct for the geometric distortion using the T2-weighted image as the anatomical reference. The deformation that relates the baseline b0 image without diffusion weighting to the T2-weighted image characterized the geometric distortion. Hence, intra-subject registration was first performed using FSL’s linear transformations (rotations and translations) between DWI and T2-weighted image. We then employed the brain warping method, large deformation diffeomorphic metric mapping (LDDMM) [48], to find the optimal nonlinear transformation that deformed the baseline image without the diffusion weighting to the T2-weighted image. This diffeomorphic transformation was then applied to DWIs in order to correct the nonlinear geometric distortion. To estimate the structural connectivity strength among cortical regions, Bayesian probabilistic tractography algorithm [55] was applied to all the seed voxels by sampling 1000 streamlines per voxel. Seed voxels were selected for the probabilistic tractography as the border voxels between cortical regions and the white matter. Fibers shorter than 10mm or looping fibers (fibers that return to the same region) were excluded from the analysis.

For each subject, whole-brain undirected weighted networks were created as follows: The connection weight (*A*_*ij*_) from the region *i* to another region *j* was defined by the following equation,

$$A_{ij}=\frac{2{Fcount}_{ij}}{C_{i}+V_{j}}$$

Where *Fcount*_*ij*_ is the number of fibers passing through the region *j* from the seed region *i*, *C*_*i*_ is the total number of fibers sampled from *i* (the multiplication of the number of voxels in region *i* with 1000 fibers per voxel based on Bayesian probabilistic tractography) and *V*_*j*_ is the total volume of region *j*. This resultant connectivity matrix is asymmetric. Thus, we used the average of *A*_*ij*_ and *A*_*ji*_ to make the final matrix *W*_*ij*_ *W*_*i j*_ symmetric (Fig 2). This is similar to the approach in [56].

### Network metrics

Neural connectivity, one of the imperative determinants of processing efficiency, has been suggested to be a potential principal mechanism underlying the concept of neural changes in brain network organization [23]. To test our hypotheses, we selected unthresholded *connectivity strength* network metric to examine age-related changes in anterior-posterior connectivity. *Connectivity strength* between two cortical regions *i* and *j* is defined as the edge weight between *i* and *j*, i.e. *wij*. Higher connectivity strength indicates stronger interconnectivity between the given regions. To streamline the number of statistical analysis needed to investigate aforementioned hypothesis, we grouped the anatomical regions of our network into a coarser level of anatomical lobes (Fig 1B and Table 2) using references in grouping [57, 58]. Additionally, as there is distinctive age-related effect on medial temporal lobe [59–61], we had further grouped the temporal sub-regions into lateral and medial temporal lobes respectively. Therefore in examining age-related changes in connectivity at level of lobes, *connectivity strength* between two lobes is defined as the average weight of connections between the regions of the corresponding lobes. And in examining age-related specific changes between individual PFC and posterior lobes, connectivity strength is defined by average weight between the PFC region and the regions of the posterior lobe.

### Statistical analysis

Linear regression analysis was performed to investigate age effects on the connectivity strength between the PFC and posterior brain regions and among the posterior brain regions at the level of lobes as well as that between individual PFC structures and the posterior brain regions. In the full regression model, the linear and quadratic terms of age were entered as the main factors and connectivity strength was as the dependent variables (connectivity strength ~ *β*_0_ + *β*_1_ *Age* + *β*_2_ *Age* ^2^ +*β*_3_ *Gender* + *ε*). In the reduced regression model, only linear term of age was entered as main factor (connectivity strength ~ *β*_0_ + *β*_1_ *Age* + *β*_2_ *Gender* + *ε*). Gender was considered as covariate in all the analyses. Bonferroni correction was carried out to correct for multiple comparisons.

To further examine whether age-related changes in the PFC connectivity strength are mediated through age-related changed in the posterior connectivity strength, the posterior connectivity strength was entered into the aforementioned regression model. Lastly, in examining whether brain atrophy would account for age-related alterations in the connectivity strength, cortical thickness was also entered into the above regression model. The Sobel-Goodman test was further used to test whether a mediator (the posterior connectivity strength or cortical thickness) carries the influence of age to the PFC connectivity strength. All analysis was performed using SPSS 18 for Windows 7.

## Results

### Age effects on brain functional connectivity

**Fig 3:**
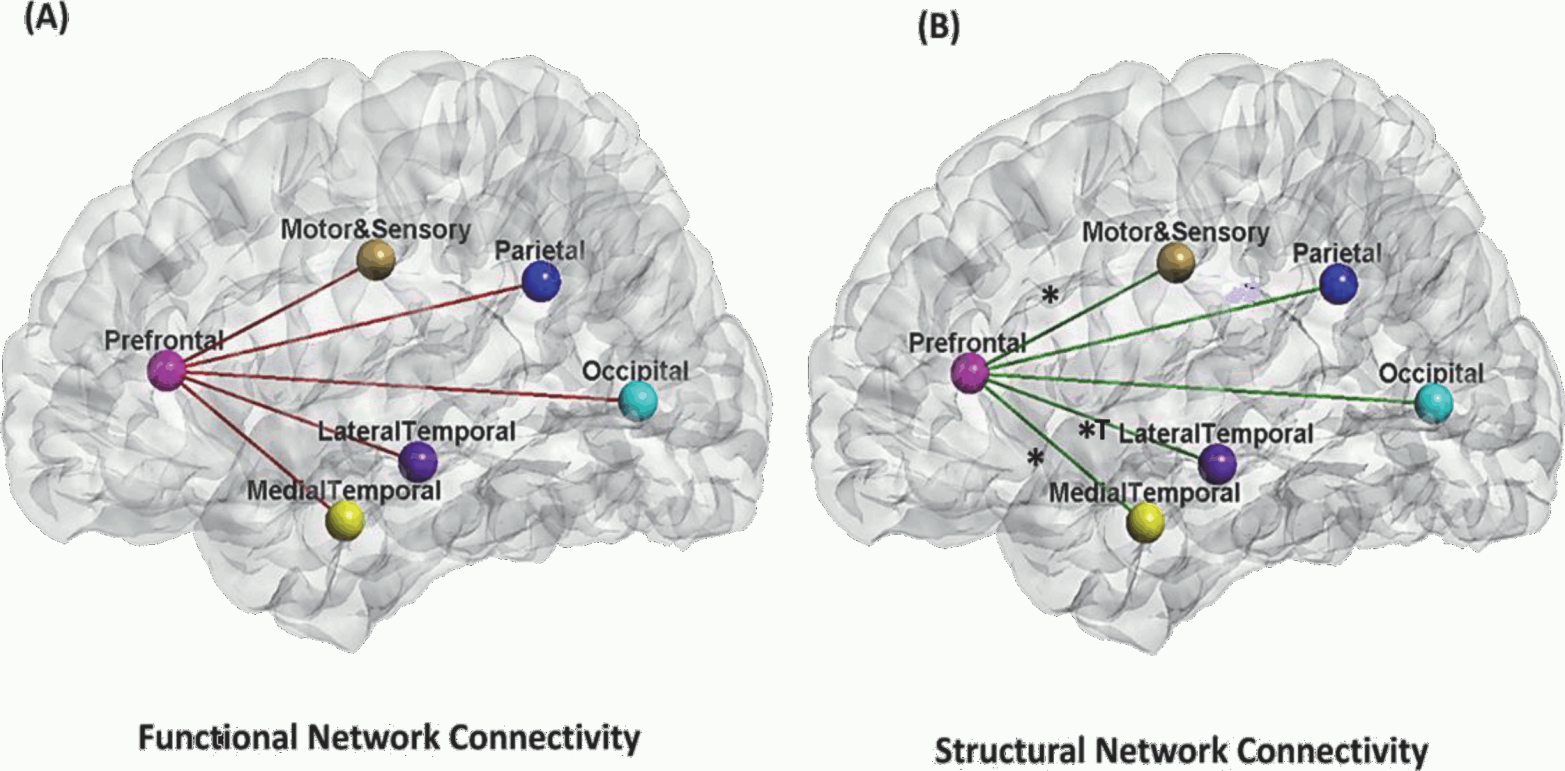
Age effects on functional (panel A) and structural connectivity (panel B) of the prefrontal cortex with other brain regions. *Note*. * denotes significant age effects on the connectivity strength between the two regions that the line connects. ^T^ denotes the significant age-related relationship even after controlling for cortical thickness.

Our analysis did not reveal age effects on the functional connectivity of the PFC with the resting brain regions at the level of brain lobes, including the lateral and medial temporal lobes, parietal lobe, motor and sensory cortex, as well as occipital lobe (the second column in Table 3; Fig 3A). However, our analysis did reveal age effects on the functional connectivity of individual PFC structures with the rest brain regions. There was an age-related decrease in the functional connectivity between the rostral middle frontal and parietal cortex (ß = -0.238, p = 0.002). After controlling for the cortical thickness for these regions, the functional connectivity between the rostral middle frontal and parietal cortex was no longer influenced by age.

**Table 3:** Linear age effects on functional and structural connectivity strength between the prefrontal cortex (PFC) and other brain regions.

Prefrontal and posterior regions connectivity at lobes level	Functional Network ß-value (*p*-value)	Structural Network ß-value (*p*-value)
PFC-Lateral Temporal	-0.144 (0.061)	0.287 (<0.001)* ^T^
PFC-Medial Temporal	-0.103 (0.180)	0.281 (<0.001)*
PFC-Parietal	-0.198 (0.010)	0.102 (0.176)
PFC-Occipital	0.114 (0.144)	0.113 (0.137)
PFC-Motor & Sensory	-0.119 (0.124)	0.229 (<0.001)*

Standardized ß-values and their corresponding p-values are listed.

*p < 0.01 (Bonferroni corrected threshold).

^T^ denotes the significant relationship between age and PFC connectivity strength even after controlling for cortical thickness of the corresponding regions.

When examining the functional connectivity of the occipital cortex with the rest of posterior brain cortices, our analysis revealed age-related linear increase in the functional connectivity strength between the occipital and lateral temporal cortices, suggesting greater functional connectivity strength between the two posterior regions in older adults (the second column in Table 4). There were no age effects on the functional connectivity of the occipital cortex with the other posterior brain regions (medial temporal and parietal cortices) (the second column in Table 4).

**Table 4:** Linear age effects on the functional and structural connectivity strength between the occipital cortex and the rest of the posterior brain cortices.

Occipital and posterior regions connectivity at lobes level	Functional Network ß-value (*p*-value)	Structural Network ß-value (*p*-value)
Occipital-Lateral Temporal	0.161 (0.036)*	0.421 (<0.001)* ^T^
Occipital-Medial Temporal	0.017 (0.822)	0.142 (0.065)
Occipital-Parietal	0.147 (0.057)	0.214 (0.002)* ^T^

Standardized ß-values and their corresponding *p*-values are reported below.

*p < 0.01.

^T^ denotes the significant relationships between age and posterior regions connectivity even after controlling for cortical thickness of the corresponding regions.

### Age effects on structural network connectivity

We first examined the structural connectivity of the PFC with the rest of the brain.

Our analysis revealed negative quadratic effects of age (inverted U-shaped) on the structural connectivity between the PFC and the parietal cortex (ß = -1.516, p = 0.003), suggesting an age-related increase in structural connectivity during early stage of adulthood and decrease structural connectivity in later life. Our results also revealed an age-related linear increase in the structural connectivity strength of the PFC with the lateral and medial temporal cortex as well as motor and sensory cortex, suggesting greater structural connectivity strength among these regions in the older adults (the third column in Table 3; Fig 3B). As the cortical thickness of the PFC and other brain regions decreases with age (p<0.001; S1 Table, the cortical thickness was further entered in the regression analysis. The structural connectivity of the PFC with the medial temporal cortex and motor and sensory cortex was no longer influenced by age after controlling for the thickness of the corresponding regions. This suggested that the cortical morphology in the medial temporal lobe and motor and sensory lobe mediates the age effects on their structural connectivities with the PFC. However, the result of the age effects on the structural connectivity between the PFC and lateral temporal cortex remained significant even after controlling for the cortical thickness of the corresponding brain regions. Lastly, like the results drawn from the functional network analysis reported above, the structural network analysis did not reveal any age effect on the structural connectivity strength between the PFC and the occipital and parietal cortices (the third column in Table 3; Fig 3B).

In examining the structural connectivity of the occipital cortex with the rest of the posterior brain cortices, there were age-related increases in the structural connectivity strength of the occipital cortex with the lateral temporal and parietal cortices, suggesting greater structural connectivity strength of the occipital cortex with the parietal and lateral temporal cortex in older adults (the third column in Table 4). These findings remained significant after controlling for the cortical thickness of the corresponding regions. The correlation of structural connectivity between the PFC and posterior regions with the cortical thickness was presented in S2 Table.

Sobel-Goodman tests further revealed that the age-related increases in the structural connectivities of the PFC with the lateral temporal cortex were partially mediated by the age-related increase in the structural connectivity of the occipital cortex with the lateral temporal cortex (z = 2.717, p = 0.006).

In the further investigation of age effects on the structural connectivity of the individual PFC structures with the temporal and parietal cortices, our results revealed negative quadratic effects of age on the structural connectivity of the ACC with the parietal cortex (ß = -1.650, p<0.001). There was also a linear age-related increase in the structural connectivity of the lateral temporal cortex with the lateral orbitofrontal (ß = 0.208, p = 0.004), medial orbitofrontal (ß = 0.235, p = 0.002), superior frontal (ß = 0.291, p<0.001), rostral middle frontal (ß = 0.229, p = 0.003) and anterior cingulate cortices (ß = 0.248, p = 0.001); the medial orbitofrontal cortex (ß = 0.321, p<0.001) with the medial temporal cortex. After controlling for the cortical thickness of these regions, the structural connectivities of the medial orbitofrontal, superior frontal and rostral middle frontal cortex with the lateral temporal cortex and between the medial orbitofrontal and the medial temporal cortex were no longer influenced by age. This again suggested that the cortical morphology in these regions, particularly the temporal cortices, mediates the age effects on the structural connection with the individual PFC regions.

## Discussion

The results from our study revealed age-related alterations in the functional and/or structural connectivity of the PFC with the posterior regions of the brain, suggesting that the brain is functionally and/or structurally well equipped to adapt to neural challenges in aging. Second, we found age-related increases in the functional and/or structural connectivity of the occipital lobe with the posterior regions of the brain, possibly suggesting reduced selectivity in neural responses within specific posterior regions with aging. Third, the age-related PFC findings were partially mediated by age-related increases in the structural connectivity of the occipital lobe within the posterior regions of the brain, suggesting that the reorganization of the PFC structural connectivity with aging could be partly due to the adaptation to age-related changes in the reorganization of the posterior regions of the brain. This thus supports the idea derived from task-based fMRI that the PFC reorganization in aging may be adapted to the need of compensations for resolving less distinctive stimulus information from the posterior brain regions. Finally, our results suggested that the structural connectivity of the PFC with the lateral temporal lobe was fully mediated by the morphology of the temporal lobe.

The structural network analyses in our study showed complementary findings supporting the idea that the reorganization of the PFC structural connectivity is adapted to neuronal challenges in aging. Our study showed age-related increases in structural connectivity of the PFC with the sensorimotor and temporal using HARDI, which is in the conjunction with the recent finding obtained using DTI, that is, an age-related increase in the frontal regional efficiency [40]. Moreover, an age-related increase in the structural connectivity between the PFC and the temporal lobe is largely consistent with task-based fMRI findings. Daselaar et al. and Dennis et al. showed that older adults had increased functional connectivity between the frontal and temporal regions during memory processing compared to young adults [8, 9]. Frey and Petrides revealed that the orbitofrontal-medial temporal lobe connection is needed for memorization of information [62]. Depending on task difficulty, various frontal areas could be engaged thus denoting an increase in the recruitment of brain regions for successful performance [63, 64]. Our finding on the structural connectivity may suggest that older adults could depend on connections between the PFC and the temporal cortex in order to successfully perform cognitive processes as compared to young adults.

Surprisingly, our study revealed age-related increases in the functional and structural connectivity of the occipital cortex with the other posterior regions. This was not observed in existing task-based fMRI studies that showed an age-related shift from stronger functional connectivity among the posterior brain regions in young adults to stronger connectivity between the posterior regions and the PFC regions in older adults [10, 14, 17]. However, Meunier et al [65] used rs-fMRI and modularity analysis and demonstrated that the number of connections of the occipital region with the parietal and temporal regions was increased in older adults. The diverse connections of the occipital lobe with the parietal and temporal lobes could be a possible reason that interprets dedifferentiation of stimuli in the posterior region of the brain in older adults [2, 8, 9, 11–13].

Interestingly, our findings supported partial mediation effects of the connectivity between the occipital lobe and the lateral temporal lobe on the connectivity between the PFC and the posterior regions of the brain. This suggested that the reorganization of the PFC structural connectivity with aging could be partly due to the adaptation to age-related changes in the structural reorganization of the posterior regions of the brain [22]. These patterns appear to be consistent with the Scaffolding Theory of Aging and Cognition (STAC) model of aging and neural adaption, which was proposed by Park and Reuter-Lorenz [22]. STAC emphasizes a process that results in changes in the brain function through strengthening of existing connections, formation of new connections, and disuse of connections that have become weak or faulty. The PFC is thought of as a locus for scaffolding and functions as a facilitative role through compensating for reduced functional specificities of the posterior brain in older adults. This has been demonstrated mostly in task-based fMRI studies [22, 66, 67]. Older adults processed stimuli in a dedifferentiated manner in the sense that functional activity of the occipital cortex extended to the other posterior regions as well as to other modalities, such as auditory modality [68]. This phenomenon was coupled with the recruitment of additional frontal processing in older adults in order to process less distinctive stimulus information [22, 66, 67, 69].

Lastly, it has been well known that age-related cortical thinning is widespread across the primary and association cortex [70]. Nevertheless, it is rarely studied how brain atrophy in aging would account for the aforementioned age-related changes in structural and functional connectivity. Our results revealed that the morphology of individual structures, especially those in the temporal lobe, fully mediated age effects on their structural network connectivity with the PFC. Structural imaging studies have consistently revealed age-related vulnerability and volume reduction in the medial temporal lobe (MTL) structures [71]. Taken together, age effects on the structural connectivity between the PFC and the temporal lobe is not beyond those on temporal lobe atrophy, suggesting that the structural recruitment of compensatory mechanism is offset by regional brain atrophy. This also highlights the importance of taking morphological measures into consideration when examining the brain networks as a function of age.

Even though the functional network examined using resting-state fMRI reflects direct and indirect anatomical connections, there is lack of direct one-to–one mapping between functional and structural brain networks [72–74]. Hence, we do expect that age could have independent effects on structural and functional networks. Moreover, [75] further suggested that age-related increases in functional connectivities for functional compensation or reconfiguration in aging may be achieved through intact underlying anatomical infrastructure.

In summary, our study employed advanced multi-modal MRI techniques, including structural MRI, rs-fMRI, and HARDI, and attested to the value of a novel multimodal combination of cortical thickness, functional and structural connectivity in aging research. The findings in our study implicated that rs-fMRI and HARDI graph analyses can replicate the task-based fMRI findings of age-related increases in PFC functional activations and age-related dedifferentiation of stimuli in the posterior regions of the brain at the level of functional and structural connectivity. Brain morphology also plays an important role in functional and structural reorganization of the brain with aging.

## Supporting Information

S1 Table Age effects on the thickness of Prefrontal and other brain regions. Standardized ß-values and their corresponding *p*-values are listed. *p < 0.01 (Bonferroni corrected threshold).
(DOCX)

S2 Table Correlation between Prefrontal and other brain regions structural connectivity and thickness. R values and their corresponding *p*-values are reported below. *p < 0.001(Bonferroni corrected threshold).
(DOCX)
