# Supplementary material for: Adaptation of Brain Functional and Structural Networks in Aging
Source: PLoS One. 2015 Apr 15;10(4):e0123462. doi: 10.1371/journal.pone.0123462 (PMC4398538; doi:10.1371/journal.pone.0123462)
Supplement: S1 Table — Standardized ß-values and their corresponding p-values are listed. *p < 0.01 (Bonferroni corrected threshold). (DOCX) [file pone.0123462.s001.docx]

**S1 Table.** **Age effects on the thickness of Prefrontal and other brain regions. Standardized ß-values and their corresponding *p*-values are listed.**

| Thickness at lobes level | ß-value (*p*-value) |
| --- | --- |
| Prefrontal | -0.255 (<0.001) |
| Lateral Temporal | -0.601 (<0.001) |
| Medial Temporal | -0.475 (<0.001) |
| Parietal | -0.621 (<0.001) |
| Occipital | -0.479 (<0.001) |
| Sensory Motor | -0.472 (<0.001) |
