# Supplementary material for: Adaptation of Brain Functional and Structural Networks in Aging
Source: PLoS One. 2015 Apr 15;10(4):e0123462. doi: 10.1371/journal.pone.0123462 (PMC4398538; doi:10.1371/journal.pone.0123462)
Supplement: S2 Table — R values and their corresponding p-values are reported below. *p < 0.001(Bonferroni corrected threshold). (DOCX) [file pone.0123462.s002.docx]

**S2 Table*.*** **Correlation between Prefrontal and other brain regions structural connectivity and thickness. R values and their corresponding *p*-values are reported below.**

|  | Prefrontal Thickness | Lateral Temporal Thickness | Medial Temporal Thickness | Parietal Thickness | Occipital Thickness | Sensory Motor Thickness |
| --- | --- | --- | --- | --- | --- | --- |
| Prefrontal-Lateral Temporal Connectivity | 0.025 (0.748) | -0.163 (0.033) | -0.399 (<0.001)* | -0.164 (0.032) | -0.142 (0.065) | -0.187 (0.014) |
| Prefrontal-Medial Temporal Connectivity | -0.124 (0.106) | -0.273 (<0.001)* | -0.291 (<0.001)* | -0.270 (<0.001)* | -0.230 (0.003) | -0.224 (0.003) |
| Prefrontal-Parietal Connectivity | -0.016 (0.839) | 0.016 (0.834) | -0.277 (<0.001)* | 0.001 (0.996) | 0.038 (0.622) | -0.101 (0.192) |
| Prefrontal-Occipital Connectivity | -0.002 (0.977) | -0.073 (0.344) | -0.140 (0.067) | -0.001 (0.991) | -0.072 (0.350) | -0.058 (0.449) |

**p* < 0.001(Bonferroni corrected threshold).
